# The Na/K-ATPase Signaling and SGLT2 Inhibitor-Mediated Cardiorenal Protection: A Crossed Road?

**DOI:** 10.1007/s00232-021-00192-z

**Published:** 2021-07-23

**Authors:** Jiang Liu, Jiang Tian, Komal Sodhi, Joseph I. Shapiro

**Affiliations:** 1 Department of Biomedical Sciences, JCE School of Medicine, Marshall University, Huntington, WV USA; 2 Department of Surgery, JCE School of Medicine, Marshall University, Huntington, WV USA; 3 Departments of Medicine, JCE School of Medicine, Marshall University, Huntington, WV USA

**Keywords:** SGLT inhibitor, Na/K-ATPase, ROS, Signaling

## Abstract

In different large-scale clinic outcome trials, sodium (Na^+^)/glucose co-transporter 2 (SGLT2) inhibitors showed profound cardiac- and renal-protective effects, making them revolutionary treatments for heart failure and kidney disease. Different theories are proposed according to the emerging protective effects other than the original purpose of glucose-lowering in diabetic patients. As the ATP-dependent primary ion transporter providing the Na^+^ gradient to drive other Na^+^-dependent transporters, the possible role of the sodium–potassium adenosine triphosphatase (Na/K-ATPase) as the primary ion transporter and its signaling function is not explored.

**Graphic Abstract:**

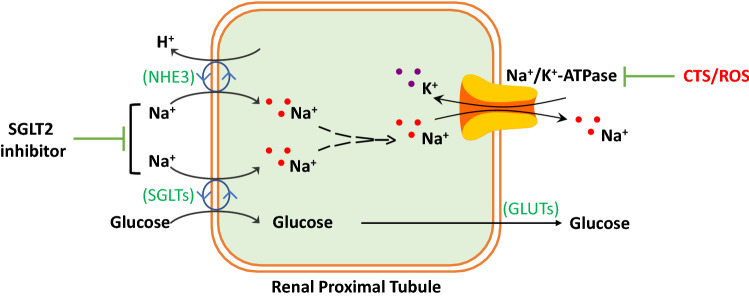

## Introduction

SGTL2 inhibitors were initially developed to lower plasma glucose in patients with type 2 diabetic mellitus (T2DM). More available data from completed clinical trials show profound cardiorenal protection in diabetic and non-diabetic chronic kidney disease (CKD) patients, which cannot be directly explained by improved glucose control. More and more insightful theories are proposed to explain the "off-target" but incredible effects of SGLT2 inhibitors other than the designed glucose-lowering property initially, as reviewed in more detail elsewhere. The molecular mechanism(s) to delineate the cardiorenal protection of SGLT2 inhibitors are still not fully understood. Based on clinical trials and experimental findings, the proposed theories include, but not limited to, the SGLT2 inhibitor-mediated regulations of (1) blood pressure through glomerular filtration rate, tubuloglomerular feedback, sodium/hydrogen (Na^+^/H^+^) exchangers, natriuresis/osmotic diuresis; (2) oxidative stress by oxygen consumption/inflammatory cytokines, as well as (3) metabolic profile alteration, growth factors, fibrotic mediators, nutrient deprivation, weight loss, ketogenesis, artery stiffness, sympathetic nervous system, and others. However, the protection of SGLT2 inhibitors could not be fully explained by a single mechanism since different clinic treatment strategies targeting different conditions are short of the overall effect of the SGLT2 inhibitors. The effects of the SGLT inhibitors are more likely related to their renal effects (Bertero et al. 2017; Gerich, 2010; Nikolic et al. 2021; Patel et al. 2021; Santos et al. 2020; Sen and Heerspink, 2021; Thomson and Vallon 2019), and an interplay of modest beneficial effects from different systems. Besides more available data from clinical trials, experiments of molecular mechanisms are much more desired. With mounting evidence of mechanisms and theories, the involvement of the Na/K-ATPase is only described as an active Na^+^/K^+^ transporter to maintain the Na^+^ gradient across the membrane, functioning as a driving force for Na^+^ and glucose reabsorption. The Na/K-ATPase also acts as a signaling transducer (coupling with tyrosine kinase c-Src) to execute different functions. This review explores the possible role of Na/K-ATPase and its signaling function that might affect SGLT2 and SGLT2 inhibitors.

## The Biology of the Na/K-ATPase

The Na/K-ATPase belongs to the P-type ATPase family and consists of two non-covalently linked α and β subunits. Several α and β isoforms, expressed in a tissue-specific manner, have been identified and functionally characterized (Blanco and Mercer 1998; Kaplan 2002; Sanchez et al. 2006; Sweadner 1989). Since J.C. Skou's discovery in 1957 (Skou 1957), the energy-transducing Na/K-ATPase has been extensively studied for its ion pumping function and, later on, its signaling role (Aizman and Aperia 2003; Shapiro and Tian 2011; Xie and Cai 2003; Zhang et al. 2019a). Cardiotonic steroids (CTS, also known as digitalis-like substances) are specific Na/K-ATPase inhibitors and ligands. CTS has been used to treat heart failure for over 200 years through its inotropic effect due to partial inhibition of Na/K-ATPase induced intracellular Na^+^ change coupled with increases in intracellular calcium (Ca^2+^) through Na^+^/Ca^2+^ exchanger (NCX). CTS also stimulates the signaling function of the Na/K-ATPase, which has been contributed to cardiac hypertrophy and fibrosis, Na^+^ reabsorption in renal proximal tubule cells (RPTs), systemic oxidative stress, and release of inflammatory cytokines. "The third factor" or "natriuretic factor", other than glomerular filtration rate and aldosterone, was postulated to regulate renal Na^+^ handling through Na/K-ATPase (Bricker 1967; Dahl et al. 1969; de Wardener and MacGregor 1980). Identification of ouabain-like substance in human plasma and in vivo studies with genetically modified mouse models (humanized ouabain-sensitive Na/K-ATPase α1) have unequivocally demonstrated that endogenous CTS regulates renal Na^+^ excretion and blood pressure through the Na/K-ATPase (Dostanic-Larson et al. 2005; Dostanic et al. 2005; Hamlyn et al. 1991; Loreaux et al. 2008).

CTS has been classified as a new class of hormones, making Na/K-ATPase a potential therapeutic target for cardiac and renal diseases (Aperia 2007; Bagrov and Shapiro, 2008; Bagrov et al. 2009; Schoner 2002; Yatime et al. 2009). CTS includes plant-derived glycosides such as digoxin and ouabain and vertebrate-derived aglycones such as bufalin and marinobufagenin. The production and secretion of ouabain and marinobufagenin can be regulated by multiple stimuli, including angiotensin II and adrenocorticotropic hormone (ACTH) (Bagrov et al. 2009; Hamlyn et al. 1991; Laredo et al. 1997; Schoner 2002; Schoner and Scheiner-Bobis 2007a, b, 2008). Endogenous CTS are present in measurable amounts under normal physiological conditions and are markedly increased under several pathological conditions such as Na^+^ imbalance, chronic renal failure, hyperaldosteronism, hypertension, congestive heart failure, plasma volume, blood pressure, and salt sensitivity (Blaustein 1993; Fedorova et al. 1998, 2001,2002; Gottlieb et al. 1992; Hamlyn et al. 1991, 1998; Hasegawa et al. 1987; Komiyama et al. 2005; Manunta et al. 2006a,^1999^; Rossi et al. 1995). studies have also revealed many extra-cardiac actions, such as in response to salt loading and hypertensions (Fedorova et al. 2005; Ferrari et al. 2006; Haddy and Pamnani 1998; Kaunitz 2006; Manunta et al. 2006b). Also, low doses of CTS induced hypertension in rats and caused significant cardiovascular (CV) remodeling independent of their effect on blood pressure (Ferrandi et al. 2004; Jiang et al. 2007; Kennedy et al. 2006; Skoumal et al. 2007).

## The Biology of SGLTs

The kidney is involved in regulating glucose homeostasis (Bergman and Drury 1938) and is critical in developing and managing diabetes mellitus, including gluconeogenesis to release glucose, glucose uptake for energy supply, and glucose reabsorption (Gerich 2010). The glucose reabsorption from glomerular filtrate in RPTs is an energy‐requiring process that reabsorbs ~ 180 g per day through SGLTs. The SGLTs belong to a structural class of membrane proteins (Bell et al. 1990; Wright et al. 2011). In RPTs, SGLTs at the apical membrane mediate the entry of glucose, and glucose transporters (GLUTs) at the basolateral membrane mediate the extrusion of glucose into the circulation. The driving force is the energy-dependent Na/K-ATPase which also extrudes reabsorbed Na^+^ into circulation. SGLT2 is a high‐capacity, low‐affinity transporter (co-transport Na^+^ and glucose at 1:1 stoichiometry) located on the S1 and S2 segments, reabsorbing ~ 90% filtered glucose. SGLT1 is a high‐affinity, low‐capacity transporter (co-transport Na^+^ and glucose at 2:1 stoichiometry) located on the S3 segment, reabsorbing the ~ 10% filtered glucose (unabsorbed glucose by SGLT2) (Brown 2000; Hediger and Rhoads 1994; Lee et al. 2007; Wright et al. 2007,2011). In healthy individuals with normal kidney function, urine is essentially free of glucose since all glomerular filtrated glucose is reabsorbed. Changes in glucose or Na^+^ filtered rate modulates the glucose transporter's (SGLT1/2 and GLUT1/2) gene expression (Vestri et al. 2001). Compared with healthy individuals, T2DM patients show significantly higher SGLT2 expression and activity, glucose reabsorption, and a higher threshold for glucosuria (Rahmoune et al. 2005; Tentolouris et al. 2019).

## Protection Effects of SGLT2 Inhibitor on Renal Disease

Data from CV outcome trials showed that SGLT2 inhibitors slow the progression of kidney function decline and reduce the risks of kidney outcomes in T2DM patients with preserved kidney function (Mosenzon et al. 2019; Neal et al. 2017a; Wiviott et al. 2018; Zinman et al. 2015a). In three outcome trials assessing the SGLT2 inhibitors in CKD patients with diabetic and non-diabetic CKD (CREDENCE trial with canagliflozin, DAPA-CKD trial with dapagliflozin, and SCORED trial with sotagliflozin), significant renal-protective outcomes were observed (Bhatt et al. 2020; Heerspink et al. 2020; Mosenzon et al. 2019; Neal et al. 2017a; Perkovic et al. 2019; Wiviott et al. 2018; Zinman et al. 2015a). Canagliflozin and dapagliflozin significantly reduced the risk of kidney failure and CV events. However, there is no significant effect in the SCORED trial, probably because of the trial's early ending. Notably, the DAPA-CKD trial demonstrated that dapagliflozin reduces the risks of major adverse kidney and CV events and all-cause mortality in diabetic and non-diabetic CKD patients with or without T2DM (Heerspink et al. 2020; Wheeler et al. 2021). In the CREDENCE trial, canagliflozin treatment reduces the risk of anemia, an independent predictor of renal and CV outcomes (Oshima et al. 2020). Furthermore, canagliflozin reduces CV events in patients with T2DM and diabetic kidney disease (DKD) and slows DKD progression. SGLT2 inhibitors also reduce the risk of new-onset diabetic nephropathy, slow the rate of kidney function decline, and reduced the risk of major kidney events (Heerspink et al. 2020; Neal et al. 2017a; Wanner et al. 2016). In patients with heart failure and reduced ejection fraction with and without T2DM, the DAPA-HF trial (Dapagliflozin and Prevention of Adverse Outcomes in Heart Failure) demonstrated that the SGLT2 inhibitor reduces the risk of heart failure hospitalizations or CV death and slow the progression of kidney function decline (Jhund et al. 2021; Packer et al. 2020). In the DAPA-HF trial, the SGLT2 inhibitor-mediated decrease in eGFR and increase in hematocrit are more likely independent of glycemia in people with and without diabetes (Lopaschuk and Verma 2020). SGLT2 inhibitor mediated a modest increase in hematocrit that can be explained by volume depletion and increased erythropoietin production (Mazer et al. 2020; Zinman et al. 2015b).

SGLT2 inhibitors usually cause weight loss in diabetic patients, mainly due to increased natriuretic/osmotic diuresis in the early treatment. However, the permanent loss of extracellular water does not occur under SGLT2 inhibition. In the long term, it mainly involves the glycosuria-caused-negative caloric balance mediated reduction of visceral/subcutaneous fat and epicardial fat mass accompanying reduced inflammatory cytokine production (Filippatos et al. 2019; Vallon and Thomson 2017). Furthermore, SGLT2 inhibition also has hepatoprotective effects by reducing fatty liver content and improve liver biology in patients with T2DM and non-alcoholic fatty liver disease (Scheen 2019; Schork et al. 2019). A metabolic shift might contribute to improving the cardiometabolic risks in T2DM patients.

Hypertension is a significant risk factor for progressive kidney function loss. SGLT2 inhibitors exert antihypertensive effects on both systolic and diastolic blood pressure without inducing a compensatory increase in heart rate (Shaikh 2017; Tikkanen et al. 2016). This phenomenon is attributed to a decrease of 30% to 60% in Na^+^ reabsorption in PRTs, improved natriuresis and diuresis, weight loss, and improved vascular function (Lopaschuk and Verma 2020; Nikolic et al. 2021). Again, the blood-pressure-lowering effects of SGLT2 inhibition are modest and cannot fully explain the beneficial CV and kidney effects.

In the CREDENCE trial, SGLT2 inhibitor canagliflozin reduces blood pressure independent of starting blood pressure levels and other concomitant blood pressure-lowering agents in patients with T2DM and CKD (Ye et al. 2021). In the DAPASALT Trial with standardized sodium diet control in T2DM patients and preserved kidney function, dapagliflozin reduced blood pressure without apparent changes in urinary Na^+^ excretion, natriuresis, and plasma volume within the period of the 2-week treatment. Interestingly, a significant increase in fractional lithium excretion was observed, indicating an increase in RPTs-mediated Na^+^ excretion that could be counteracted by a downstream compensatory mechanism (Scholtes et al. 2021).

## Protection Effects of SGLT2 Inhibitor on Cardiovascular Disease (CVD)

Inhibition of SGLT2 has been shown to improve CV outcomes in patients with diabetic kidney disease and patients without diabetics. The EMPA-REG OUTCOME study (Zinman et al. 2015c) was the first clinical trial to mark success for secondary and tertiary CVD prevention with an SGLT2 inhibitor. The study found that empagliflozin was associated with a significant reduction in mortality, heart failure hospitalization, and kidney disease progression. It was found no significant difference in the risk of stroke with empagliflozin versus placebo. Results from the CANVAS trial further substantiated the preventive effect of SGLT2 inhibitors in CV outcomes. The CANVAS study included about 10,000 patients treated with canagliflozin or placebo and followed for 3.6 years. The result showed that canagliflozin treatment resulted in a lower incidence of CV death, nonfatal myocardial infarction, nonfatal stroke, and HF hospitalization (Neal et al. 2017b). The CVD-REAL study was a large multinational program to study patients with T2DM who did not have preexisting CVD (Kosiborod et al. 2017). The study showed a 51% lower risk of death and a 39% lower risk of hospitalization for HF in patients treated with empagliflozin compared to other categories of glucose-lowering drugs. It is noted that the CVD-REAL study is an observational rather than a randomize-controlled clinical trial. Several other clinical studies have shown that SGLT2 inhibitors decrease blood pressure (Baker et al. 2014; Kario et al. 2018; Vasilakou et al. 2013). In a recent meta-analysis using data from 27 clinical studies with over 7000 patients with diabetes or chronic kidney disease, SGLT2 inhibitors were found to reduce the risk of the composite CV outcome, hospitalized or fatal heart failure, and myocardial infarction. However, the analysis showed no apparent effect on stroke or CV death. It was also found that the overall risk of genital infections was increased by SGLT2 inhibition (Toyama et al. 2019). Since SGLT2 is dominantly expressed in the kidney proximal tubules, the beneficial effect of SGLT2 inhibitors on CV outcomes was mostly considered as a secondary effect of the renal function improvement and systematic glucose-lowering effect. The preserved effects of SGLT2 inhibitors on natriuresis and blood pressure may be a pivotal pathway to CV complications, especially heart failure (List and Whaley 2011; Sattar and McGuire 2018). SGLT2 inhibitors also improve renal hemodynamics and reduce the preload and afterload in heart failure patients (Cherney et al. 2014a, b; Hung et al. 2014). The metabolic effects such as lowering glycated hemoglobin, lipid profile change, and weight loss may also help reduce the CV risks (Desouza et al. 2015; Stark Casagrande et al. 2013; Wilding et al. 2013; Zinman et al. 2015c). However, due to the mild and inconsistent results in glucose-lowering by SGLT2 inhibitors, the glycemic control seems not the primary driving force for reducing CV events (Duckworth et al. 2009; Toyama et al. 2019).

## Mechanistic Perception of SGLT2 Inhibitor and Na/K-ATPase

The heart and kidney are inextricably and functionally linked, referred to as the cardiorenal syndrome (Ronco et al. 2008). For example, about 60% of HF patients have co-morbid CKD (Heywood et al. 2007; Hillege et al. 2006). Dapagliflozin causes volume depletion without an increase in the risk of hypoglycemia in non-diabetic patients, indicating the beneficial effects of SGLT2 inhibitors extend well beyond patients with T2DM. Secondary analyses of the EMPA-REG OUTCOME trial (Empagliflozin Cardiovascular Outcome Event Trial in Type 2 Diabetes Mellitus Patients) indicate that CV and kidney function benefits are unlikely mediated by the glucose-lowering properties of the SGLT2 inhibitors (Inzucchi et al. 2018). The cardiorenal syndrome is critical in HF management, which could be directly addressed by SGLT2-inhibitors (Fathi et al. 2020). As discussed above, the outcomes of different clinic trials show the co-existence of CV and renal protection, indicating the involvement of other non-glycemic pathways by SGLT2 inhibition (Bell and Yellon 2018).

Interestingly, both in vitro and in vivo mechanistic studies demonstrated some SGLT2 inhibitor-induced beneficial effects are independent of SGLT2 inhibition. Furthermore, the Na/K-ATPase and its signaling share some of the regulatory pathways by SGLT2 inhibitors, such as natriuresis, blood pressure, oxidative stress, inflammation.

### Na ^+^ Reabsorption and Natriuresis

Natriuresis leads to volume contraction and decrease in blood pressure glomerular hyperfiltration. SGLT2 is responsible for ≈5% of Na^+^ reabsorption in RPTs under normal conditions. Chronic hyperglycemia increases the expression and activity of SGLT2, leading to increased plasma volume and blood pressure. SGLT2 inhibition cause volume contraction, decreased blood pressure, and reduces glomerular pressure (Lytvyn et al. 2017). SGLT2 inhibition-mediated natriuresis is likely a major factor leading to cardiorenal protective effects observed with empagliflozin and canagliflozin, which appear to extend across CKD stages (Petrykiv et al. 2017).

SGLT2 inhibitor has been shown to cause natriuresis, volume contraction, and blood pressure lowering. RPTs osmotically reabsorb ~ 60–70% of filtered Na^+^ back to circulation. This process includes the Na^+^ entry through NHE3, SGLTs, and other Na^+^-dependent transporters located on the apical membrane, and the Na^+^ extrusion through energy (ATP)-dependent Na/K-ATPase located on the basolateral membrane. The activity of NHE1 and NHE3 is upregulated in the settings of heart failure and T2DM (Packer 2017). SGLT2 and NHE3 are co-localized on the apical membrane of RPTs and functionally affecting each other. While SGLT2 inhibitor inhibits NHE3 activity, tubular-specific NHE3 knockout mice showed reduced SGLT2 expression and reduced natriuretic effect by SGLT2 inhibitor (Thomson and Vallon 2019). Empagliflozin causes volume contraction by increasing urinary excretion of Na^+^ and bicarbonate in wild-type littermates, but not in non-diabetic mice with tubular-specific NHE3 knockdown. Moreover, in type 1 diabetic Akita mice, chronic empagliflozin treatment inhibits NHE3 activity by enhanced phosphorylation of NHE3 (S552/S605), indicating that NHE3 is a determinant of the natriuretic effect of empagliflozin (Onishi et al. 2020). When the type 1 diabetic Akita mice were compared with the type 1 diabetic Akita mice with tubular-specific NHE3 knockout, the NHE3 knockout mice show a battery of changes, which indicate that the absence of tubular NHE3 likely shifted Na^+^ and glucose reabsorption from SGLT2 to SGLT1 that is likely associated with a pro-inflammatory renal signal (Onishi et al. 2019).

Glomerular hyperfiltration is a common pathway of kidney injury both in diabetic and non-diabetic settings and is associated with the progression of kidney function decline (Helal et al. 2012). Under hyperglycemic conditions, increased RPT reabsorption of glucose and Na^+^ causes decreased distal Na^+^ delivery, leading to the activation of tubuloglomerular feedback (TGF) and glomerular hyperfiltration. SGLT2 inhibitors reduce hyperfiltration by inhibiting Na^+^ reabsorption in RPTs (Cherney et al. 2014b; Heerspink et al. 2016; Wanner et al. 2018). In Akita mice (a T1DM model), SGTL2 inhibition decreases glomerular hyperfiltration (Heerspink et al. 2016; Kidokoro et al. 2019). In T2DM patients, SGLT2 inhibition reduces the estimated glomerular filtration rate (eGFR) associated with the preservation of long-term kidney function (Wanner et al. 2018). In both T1DM and T2DM patients with normal kidney function, dapagliflozin causes an acute fall in GFR accompanied by a reduction in renal blood flow and renovascular resistance (van Bommel et al. 2020).

### Mesangial Cells

Diabetic kidney disease is the most common cause of chronic kidney disease and end-stage renal failure. Mesangial cells (MCs) play an important role in regulating glomerular filtration and in the development of diabetic nephropathy (Schena and Gesualdo 2005). Damage of MCs leads to mesangial expansion and contributes to glomerulosclerosis (Abrass 1995; Mason and Wahab 2003), and reduced contractile response of MCs is a known cause of hyperfiltration (Donnelly et al. 1996; Gnudi et al. 2007; Kreisberg, 1982). Expression of the Na/K-ATPase, NHEs, and SGLTs was demonstrated in the membrane of MCs, and SGLT2 may function as a normal physiological glucose sensor and regulate cellular contractility because of its high sensitivity to short-term high glucose exposure (Kuriyama et al. 1992; Maki et al. 2019b; Wakisaka and Nagao 2017; Wakisaka et al. 2016). Exposure of MCs to high glucose significantly increased SGLT2 expression that was attenuated by Canagliflozin or ipragliflozin. Treatment with canagliflozin and phlorizin inhibited high-glucose-induced activation of PKC-NAD(P)H oxidase pathway and PKC- TGF-β pathway to increase ROS and Type IV collagen production (Donnelly et al. 1996; Maki et al. 2019b). SGLT2 in MCs has a direct protective effect on podocytes and MCs (Maki et al. 2019b). Notably, a low dose of canagliflozin improved albuminuria and mesangial expansion in type 2 *db/db* mice without lowering the blood glucose level (Maki et al. 2019b). Furthermore, the mesangial expansion in T1D Akita mice was significantly improved by canagliflozin than by insulin (Miyata et al. 2020).

### The Na/K-ATPase Signaling Prevented Hyperglycemia-Induced Apoptosis

In primary cultures, a moderate increase in glucose concentration (10–15 mM, compared with normal physiological 5 mM) causes an SGLT-dependent apoptotic response in SGLT-expressing RPTs and MCs that was abolished by administration of SGLT inhibitor or knockdown of SGLT2 in RPTs, but not in podocytes that lack SGLTs (Nilsson et al. 2019). Most interestingly, treatment with a low concentration of a specific ligand/inhibitor of the Na/K-ATPase, ouabain (at 5 nM), prevented not only high glucose-induced apoptosis and changes in expression of Bax and Bcl-xL in RPTs and MCs, but also prevented high glucose-induced mitochondrial depolarization and increased ROS formation in RPTs (Nilsson et al. 2019). Ouabain (5 nM), which does not affect intracellular Na^+^ concentration, triggers a calcium-NF-κB signal that protects kidney development from the adverse effects of malnutrition (Li et al. 2010). Furthermore, exposure of rat RPTs to high glucose increased Na/K-ATPase activity and Na/K-ATPase-dependent energy consumption (Körner et al. 1994). These observations further emphasize the importance of the Na/K-ATPase and its signaling function. While ouabain (5 nM) rescued RPTs from apoptosis in animal models of proteinuric disease (Burlaka et al. 2016) and hemolytic uremic syndrome (Burlaka et al. 2013), ouabain also rescued both RPTs and MCs from high glucose-triggered apoptosis (Nilsson et al. 2019). The underlying mechanism of a low dose of ouabain is the rebalance of the apoptotic factor Bax and antiapoptotic factor Bcl-xL, mainly through ouabain-stimulated Na/K-ATPase/IP3R/NF-κB pathway. It will be of great interest to investigate the specific response of different types of cells from different organs since SGLT inhibitors execute various effects under different conditions.

### The Na/K-ATPase Signaling Stimulated Natriuresis

From the proposed theories, the Na/K-ATPase ion transporter and signaling function of the Na/K-ATPase may present some similar effects of SGLT2 inhibitors. Under normal condition, the proximal tubular Na/K-ATPase is the driving force to reabsorb ~ 65% of filtered Na^+^ (Boron and Boulpaep 2012; Curthoys and Moe, 2014) (~ 5% by SGLT2), ~ 100% of filtered glucose (~ 90% by SGLT2 and ~ 10% by SGLT1). NHE3 is believed to be the rate-limiting step of total RPT Na^+^ reabsorption as well as a critical regulator of acid–base equilibrium through the link between NHE3-mediated H^+^ secretion to HCO_3_^−^ reabsorption by Na^+^/HCO_3_^−^ co-transporter (Alpern 1990; Amemiya et al. 1995; Aronson 1983; Biemesderfer et al. 1993; Hamm et al. 2015). Activation of the Na/K-ATPase signaling function induces coordinated endocytosis of the Na/K-ATPase and NHE3 that leads to reduced Na^+^ reabsorption and natriuresis to cause volume contraction and blood pressure-lowering (Cai et al. 2008; Liu et al. 2004, 2005, 2011; Liu and Shapiro 2007; Periyasamy et al. 2005). This reduced Na^+^ reabsorption also reduced glomerular hyperfiltration, mimicking SGLT2 inhibitor-induced reduction of glomerular hyperfiltration by inhibiting Na^+^ reabsorption in RPTs (Cherney et al. 2014b; Heerspink et al. 2016; Wanner et al. 2018). These reflect SGLT2 inhibitor's effects on NHE3, Na^+^ homeostasis, and glomerular hyperfiltration. However, it is not clear whether and how the Na/K-ATPase signaling function affects glucose uptake through SGLTs. On the other hand, SGLT2 inhibitors reduce SGLT2-mediated Na^+^ uptake into RPTs, which favors an E2(P) conformation of the Na/K-ATPase that tends to activate the signaling function. It worth noting that ouabain-induced trafficking of the Na/K-ATPase and NHE3 is independent of intracellular Na^+^ change (Cai et al. 2008), but ouabain binding induces the E2(P) conformation to inhibit Na/K-ATPase activity. A culprit is that the Na/K-ATPase signaling may increase ROS generation, whereas the SGLT2 inhibitor reduces oxidative stress. Since the Na/K-ATPase signaling is redox-sensitive (Liu et al. 2020), overstimulated signaling may chronically desensitize the signaling function and reduce Na/K-ATPase ion-transport capability by stimulating Na/K-ATPase/c-Src endocytosis. In clinical trials with antioxidant supplements, on the other side, the beneficial effect is controversial and not seen in most of the trials (Huang et al. 2006; Munzel et al. 2010; Touyz 2004). No matter what it may be, it seems like the balance of the redox status within a physiological range may be critical to maintaining beneficial ROS signaling. This disagreement worth futural investigation.

### The Na/K-ATPase Signaling Stimulated Oxidative Stress

Other than reduced SGLT2-mediated Na^+^ uptake, both ouabain and glucose oxidase cause direct protein carbonylation of Pro^222^ and Thr^224^ residues of the Na/K-ATPase α1 subunit in porcine proximal tubule LLC-PK1 cells (Yan et al. 2013). The Pro^222^ and Thr^224^ are located in peptide ^211^VDNSSLTGESEPQTR^225^ [UniProtKB*/Swiss-Prot No P05024 (AT1A1_PIG)*], which is 100% identical amongst human, pig, rat, and mouse. The Pro^222^ andThr^224^ are located in the actuator (A) domain, highly exposed and facing the nucleotide-binding (N) domain. Upon ouabain binding, Na/K-ATPase undergoes E1(P) to E2(P) conformational change, which affects the binding of the α1 subunit to signaling molecules such as c-Src and PI3K (Yatime et al. 2011). Protein carbonylation is reversible (decarbonylation) that may function as a regulatory mechanism of cell signaling (Wong et al. 2008, 2010). An undefined decarbonylation process of the Na/K-ATPase was also observed (Yan et al. 2013) as seen in the reversed Na/K-ATPase ion-pumping activity after removing ouabain from culture medium (Liu et al. 2004). It is possible that carbonylation modification might stabilize the Na/K-ATPase in a certain conformational status favoring ouabain binding to the Na/K-ATPase **α**1 subunit and thus ouabain-Na/K-ATPase signaling.

A feed-forward, redox-sensitive Na/K-ATPase signaling-mediated oxidant amplification loop, stimulated either by CTS or ROS (Pratt et al. 2018). The Na/K-ATPase signaling and its amplification loop play an essential role in the regulation of cardiac hypertrophy, salt-sensitive hypertension in both Dahl salt-sensitive rats and polygenic obese TALLYHO/JngJ mice (a mouse model mimics the state of obesity in humans with a polygenic background of type 2 diabetes), RPT Na^+^ reabsorption, systemic redox status, experimental CKD-induced cardiomyopathy (including left ventricle hypertrophy and cardiac/renal fibrosis) and anemia (Liu et al. 2020, 2016a, 2011,2016b; Pratt et al. 2018; Sodhi et al. 2020; Wang and Shapiro 2019; Yan et al. 2019). However, it worth noting that this positive feedback mechanism might chronically desensitize the signaling function and reduce Na/K-ATPase ion-transport capability by stimulating Na/K-ATPase/c-Src endocytosis (Cai et al. 2008; Liu et al. 2004, 2005). Compared with Dahl salt-resistant rats, Dahl salt-sensitive rats have a higher oxidative background in RPTs, and a high salt diet was unable to stimulate the Na/K-ATPase signaling, natriuresis, and endocytosis of the Na/K-ATPase and NHE3 (Liu et al. 2011).

CKD patients tend to have increased circulating CTS (Kolmakova et al. 2011; Komiyama et al. 2005). In addition, in experimental animal models of 5/6th partial nephrectomy (PNx) and marinobufagenin (MBG, one of the cardiotonic steroids) infusion, similar uremic cardiomyopathy phenotypes, such as elevated circulating MBG in PNx, cardiac hypertrophy, impaired cardiac function, and cardiac fibrosis, were observed (Elkareh et al. 2007; Kennedy et al. 2003, 2006; Zhang et al. 2019b).

The Na/K-ATPase/Src/ROS feed-forward oxidant amplification loop was also demonstrated in vivo in the development of uremic cardiomyopathy and anemia in a pole ligation PNx mouse model (AuWang et al. 2017; Liu et al. 2016a), which showed renal dysfunction with cardiac hypertrophy, cardiac fibrosis, and increase protein carbonylation. Administration of pNaKtide, an antagonist of Na/K-ATPase/Src signaling that binds to c-Src kinase domain (Li et al. 2009, 2011; Li and Xie 2009), attenuated the induced uremic cardiomyopathy. It indicates that the oxidant amplification loop is critical for the development of uremic cardiomyopathy.

### Oxidative Stress and Inflammation

Oxidative stress is a well-recognized contributor in the development and progressions of diabetes/diabetic nephropathy, as well as in initiation and deterioration of cardiac structural changes and HF diabetic complications (Asmat et al. 2016; Bonventre 2012; Fine and Norman 2008; van der Pol et al. 2019). The Na/K-ATPase creates and maintains the Na^+^ gradient across the membrane, accounts for most of the kidney's oxygen consumption for reabsorption of filtered Na^+^ in RPTs (Hansell et al. 2013). The increase in cortical tubular Na/K-ATPase-related oxygen consumption in diabetic rats can be abolished by administering phlorizin, a non-selective inhibitor of SGLT 1 and 2 (Körner et al. 1994).

Oxidative stress and inflammation are essential contributors to heart failure and renal failure, and diabetic cardiac remodeling is redox-sensitive (Nikolic et al. 2021; Pickering et al. 2018; Wilson et al. 2018). A low dose of SGLT2 inhibitor canagliflozin has a renal-protective effect independent of its glucose-lowering effect but may involve inhibition of high-glucose-induced DAG-PKC activation and ROS overproduction (Maki et al. 2019a).

Upregulation of pro-inflammatory cytokines contributes to ROS generation and cardiac/renal fibrosis in DM and HF settings. In different animal models with T2DM, prediabetic metabolic syndrome, and induced diabetic cardiomyopathy, Dapagliflozin and Empagliflozin ameliorate cardiac fibrosis/remodeling and cardiac function by its anti-inflammatory, anti-ROS, and anti-fibrotic effects (Arow et al. 2020; Kusaka et al. 2016; Lin et al. 2014; Ye et al. 2017). NADPH oxidases family is one of the major mediators of ROS production. In a rabbit model of HF, antioxidant N-acetylcysteine reduces NF-κB activity and cardiomyocyte apoptosis (Wu et al. 2014). SGLT2 inhibitor Empagliflozin has been shown to inhibit NOX4 expression and activity, attenuate myocardial and renal oxidative stress/inflammation and fibrosis, decrease renal gene expression of inflammation and oxidative stress in diabetic mice and a transgenic rat model of prediabetic metabolic syndrome(Kusaka et al. 2016; Li et al. 2019; Terami et al. 2014).

SGLT2 inhibitors also improve the inflammatory and oxidative stress status in RPTs (Hatanaka et al. 2016; Panchapakesan et al. 2013; Shirakawa and Sano 2020). SGLT2 inhibitors reduce nuclear factor kappa B (NF-κB), interleukin-6 (IL-6), monocyte chemoattractant protein-1 (MCP-1), and other inflammatory factors implicated in DKD pathogenesis in experimental models of diabetes and in T2DM patients (Dekkers et al. 2018; Mancini et al. 2018). Under equal glycemic control with canagliflozin and glimepiride, only canagliflozin reduced pro-inflammatory mediators, suggesting a direct anti-inflammatory effect (Heerspink et al. 2019).

The Na/K-ATPase/Src/ROS feed-forward oxidant amplification loop has also been demonstrated to increase oxidative stress and pro-inflammatory cytokines in other types of animal models, including Western diet, obesity, aging, steatohepatitis, atherosclerosis, and adipogenesis (Pratt et al. 2019; Sodhi et al. 2017, 2018), which could be attenuated by administration of pNaKtide.

### Ca^2+^ and Na^+^ Handling

Heart failure is associated with impaired Ca^2+^ and Na^+^ handling in failing cardiac myocytes that involves NHE1 and Na^+^/Ca^2+^ exchanger (NCX) that are linked to the Na/K-ATPase (Armoundas et al. 2003; Baartscheer et al. 2003a, b, 2005; Bers and Despa 2006; Müller-Ehmsen et al. 2003; Swift et al. 2008). Cardiac SGLT1 expression is upregulated to increase Na + influx and glucose uptake both in animal models of type 2 diabetes and in cardiac tissue harvested from patients with diabetic cardiomyopathy, and its activity contributes to the increase in intracellular Na^+^ (Banerjee et al. 2009; Lambert et al. 2015). An increase in intracellular Na^+^ has been shown to increase ROS generation and related hypertrophy and fibrosis (Kohlhaas et al. 2010; Murdoch et al. 2006; Seddon et al. 2007). In isolated ventricular myocytes from rabbits and rats, Empagliflozin reduced intracellular Na^+^ and Ca^2+^ by directly inhibiting NHE, which is independent of SGLT2 activity (Baartscheer et al. 2017).

SGLT2 inhibitors shift metabolism from carbohydrates towards lipolysis, thus promoting mild ketogenesis, which may provide an alternative energy substrate to myocardial cells in the setting of ischemic stress (Ferrannini et al. 2016). SGLT2 inhibitors block NHE1, which is upregulated in heart failure. Long-term suppression of NHE1 in animals has been demonstrated to reduce oxidative stress and thus, myocardial fibrosis and left ventricular remodeling (Prasad et al. 2013). Empagliflozin was reported to block NHE1-induced cell death, hypertrophy, and tissue damage in heart (Sakata et al. 2000). In addition, although SGLT2 is not expressed in heart tissue, SGLT1 is present in lower levels in the myocardium (Kashiwagi et al. 2015). Some SGLT2 inhibitors could block both SGLT2 and SGLT1 in *ex-vivo* experiments (Lim et al. 2019), suggesting that SGLT2 inhibitors may benefit from the inhibition of SGLT1 in cardiac tissue.

NHE1 is the main NHE isoform expressed in myocardial tissue in the heart, regulating pH and volume in cardiomyocytes (Wakabayashi et al. 2013). The NHE1 activity is upregulated in the settings of HF and T2DM (Packer 2017) that is contributed to intracellular Na^+^ and Ca^2+^ overload through coupling with the Na/K-ATPase, and NHE1 inhibition decreases cardiac remodeling, necrosis, and hypertrophy. All three SGLT2 inhibitors directly suppress the NHE1 activity in isolated cardiomyocytes (from mouse, rat, and rabbit) (Baartscheer et al. 2017; Uthman et al. 2018a), probably through direct binding to the Na^+^-binding site of NHE1 since SGLT2 is not found in heart up to now. In isolated cardiomyocytes from diabetic cardiomyopathy and diabetic animal models, Dapagliflozin and Empagliflozin reduce expression of NHE1 and NCX, and improve Ca^2+^ handling (Arow et al. 2020; Hammoudi et al. 2017; Joubert et al. 2017). Moreover, Empagliflozin maintains cell viability and ATP content following hypoxia/reoxygenation in cardiomyocytes and endothelial cells (Uthman et al. 2018b).

In cardiac-specific human NADPH oxidase 4 (Nox4) transgenic mice, an increase in NOX4 protein expression leads to an increase in ROS generation with cardiac interstitial fibrosis through activation of protein kinase B-mechanistic target of rapamycin (Akt-mTOR) and NF-κB signaling pathways (Zhao et al. 2015). The attenuation of cardiac oxidative stress and inflammation leads to weight loss, reduced subcutaneous fat mass, and visceral adipocyte size (Kusaka et al. 2016). Since there is no evidence of SGLT2 expression in the heart and SGLT1 is widely expressed in the myocardium, it further supports the idea that the cardio-protective effects of SGLT2 inhibitors might be stemmed from their renal-protective effects.

In the coupling of the function of SGLT1 and SGLT2, two other proteins, the Na/K-ATPase and glucose transporter, are important to remove the increased Na^+^ and glucose to maintain the homeostasis. However, the functional interaction between Na/K-ATPase and SGLT proteins in renal and CV diseases has not been extensively studied. The Na/K-ATPase has been known to play an essential role in both renal and CV diseases. Human hearts express three alpha isoforms (α1, α2, α3) of Na/K-ATPase (Gilmore-Hebert et al. 1989; Lucchesi and Sweadner 1991; Shamraj et al. 1991). Na/K-ATPase has been extensively studied for its ion transporting function since it was discovered in the 1950s. It was until the early 2000s, the signaling function of Na/K-ATPase started to be appreciated (Aizman and Aperia 2003; Shapiro and Tian 2011; Xie and Cai 2003; Zhang et al. 2019a).

Recent studies demonstrated that overstimulation of Na/K-ATPase signaling by increased endogenous CTS causes elevation of oxidative stress, which may play an important role in the uremic cardiomyopathy, including cardiac hypertrophy and cardiac fibrosis (Liu et al. 2020; Sodhi et al. 2020; Wang et al. 2020). In addition, low doses of CTS induced hypertension in rats and caused significant CV remodeling independent of their effect on blood pressure (Ferrandi et al. 2004; Jiang et al. 2007; Kennedy et al. 2006; Skoumal et al. 2007). Dilated cardiomyopathy patients tend to have decreased cardiac Na/K-ATPase expression (Norgaard et al. 1988; Schwinger et al. 2003). In isolated rat (neonatal and adult) cardiac myocytes, both ouabain and exogenously added glucose oxidase (or a bolus of H_2_O_2_) activate Na/K-ATPase signaling that leads to hypertrophy of cardiac myocytes (Liu et al. 2000, 2006; Tian et al. 2003; Xie et al. 1999). The Na/K-ATPase signaling is independent of intracellular Ca^2+^ and Na^+^ concentrations (Liu et al. 2000; Xie et al. 1999).

**Fig. 1:**
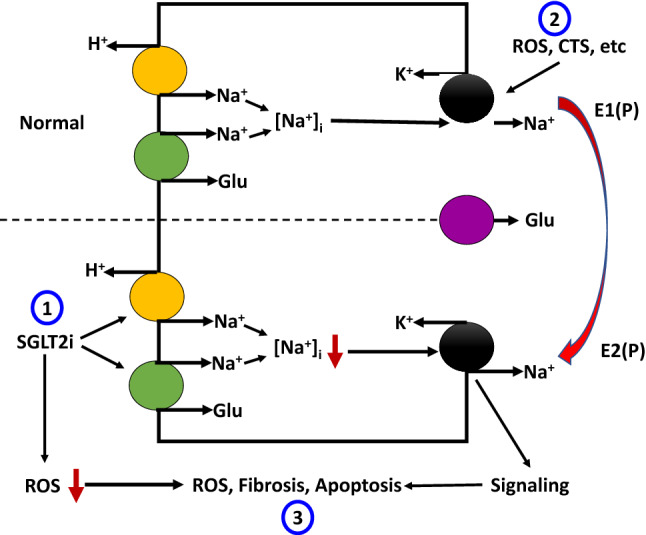
Schematic illustration of possible interactions between SGLT2 and Na/K-ATPase. (1) SGLT2 inhibitors inhibit apical Na^+^ entry through both SGLT2 and NHE3 that may account for up to ~ 70% of Na^+^ entry in RPTs, leading to lower intracellular Na^+^ concentration, and thus the Na/K-ATPase activity. This situation favors the Na/K-ATPase in an E2(P) status that favors activation of the Na/K-ATPase signaling. (2) On the other side, increases in CTS or ROS cause inhibition of the Na/K-ATPase activity that increases intracellular Na^+^ concentration, leading to inhibition of SGLT2 and NHE3. CTS or ROS also stimulates Na/K-ATPase signaling and subsequent endocytosis of the Na/K-ATPase and NHE3 and fibrotic response. (3) While activation of the Na/K-ATPase signaling increases oxidative stress and fibrosis, SGLT2 inhibitors reduce oxidative stress to counterbalance the fibrotic response. It is worth noting that a low dose of ouabain prevents hyperglycemia-induced apoptosis and ROS generation

From clinical data and animal studies, it has been demonstrated that decrease of Na/K-ATPase is an important risk factor for cardiac decompensation and dysfunction (Drummond et al. 2016, 2014; Ishino et al. 1999; Liu et al. 2012; Moseley et al. 2004; Norgaard et al. 1988; Schmidt et al. 1993; Semb et al. 1998). It has been reported that Na/K-ATPase concentration and activity were reduced in patients with heart failure, and cardiac ejection fraction was correlated with the amount of Na/K-ATPase protein level in heart tissue (Ishino et al. 1999; Moseley et al. 2004; Norgaard et al. 1988; Schmidt et al. 1993). Using the weighted gene co-expression network analysis (WGCNA) based on data from a large cohort of heart transplant patients and their donors (GEO141910), we identified the gene that includes Na/K-ATPase was significantly associated with the phenotype of dilated cardiomyopathy. More importantly, the Na/K-ATPase expression level in this cohort strongly correlates with the left ventricle ejection fraction (LVEF), consistent with the previous findings in heart transplant patients (Ishino et al. 1999; Norgaard et al. 1988; Schmidt et al. 1993). More recently, it was demonstrated that reduction of α1 isoform of Na/K-ATPase causes tissue fibrosis and cardiac cell apoptosis in response to Na/K-ATPase ligand treatment and in the animal model of CKD (Drummond et al. 2014; Liu et al. 2012). It was also found that CKD induces activation of Na/K-ATPase-mediated Src and its downstream target NFκB, which results in a reduction of miR-29b-3p expression and cardiac tissue fibrosis (Drummond et al. 2016, 2018). Disrupting the Na/K-ATPase-related signaling and inhibition of Src activation by pNaKtide increased miR-29b-3p expression in heart tissue and thus attenuated cardiac fibrosis in these animals (Drummond et al. 2018). In LLC-PK1 cells, high-glucose treatment at the basolateral side alone or basolateral plus apical sides, but not in the apical side alone, upregulates SGLT2 expression and activity via activation of the GLUT2/importin-α1/HNF-1α pathway (Umino et al. 2018). Interestingly, in cultured human proximal tubule cells, H_2_O_2_ but not high-glucose causes upregulation of SGLT2 protein expression and activity via ROS generation (Nakamura et al. 2015). SGLT2 deficiency causes glucosuria without volume depletion (Vallon et al. 2011). Proximal tubule-specific NHE3 knockout upregulates SGLT2 expression and lowers blood pressure by increasing the pressure natriuresis (Li et al. 2018). Model-based clinical data analysis indicates that NHE3 inhibition is a required mechanism for the gliflozin-induced natriuretic effect (Hallow et al. 2018). Exposure of rat proximal tubules to high glucose results in increased Na/K-ATPase activity and Na/K-ATPase-dependent energy consumption (Körner et al. 1994). Diabetic rats also showed significantly higher GFR, renal oxygen metabolism, and Na^+^ reabsorption than the control rats, as well as higher Na/K-ATPase activity in cortical tubular but not glomerular tissue. These changes were blocked by Phlorizin treatment(Körner et al. 1994). Diabetes is associated with increased renal oxygen metabolism secondary to the increase in coupled Na^+^ reabsorption via the SGLTs and Na/K-ATPase, which might contribute to the hyperperfusion and hyperfiltration in the diabetic kidney. Damage of tubular cells causes interstitial fibrosis and glomerular tubular dissociation that can be abolished by catalase overexpression (Brezniceanu et al. 2008). Apoptosis is associated with increased secretion of transforming growth factor-β (TGF-β) and other proinflammatory cytokines that drive the fibrotic process (Meng et al. 2016; Ramesh et al. 2009), it was proposed that apoptotic responses of RPTs to hyperglycemia are a major cause of the progressive interstitial fibrosis in DKD (Nilsson et al. 2019). On the other hand, activation of the Na/K-ATPase-Src signaling pathways also increased ROS generation and fibrosis in kidney and heart in 5/6^th^ PNx mouse/rat model and Dahl salt-sensitive hypertensive rat model (Haller et al. 2012; Liu et al. 2016a; Zhang et al. 2019b), which might be contributed to the activation of TGF-β, mammalian target of rapamycin (mTOR), and PLC/PKC-*δ* pathway, which induced phosphorylation and degradation of transcription factor Friend leukemia integration-1 (Fli-1, a negative regulator of collagen synthesis) (Elkareh et al. 2007, 2009; Haller et al. 2016; Zhang et al. 2019b). In these models, fibrotic responses to PNx surgery and high salt diet challenge were significantly attenuated by administration of pNaKtide (a specific peptide to block Na/K-ATPase-Src signaling) or monoclonal antibody against MBG (Fig. 1).

## Conclusion

Other than the glucose-lowering property as initially designed, SGLT2 inhibitors show profound "off-target" but beneficial cardiorenal-protective effects, promoting mechanism investigations and theories. The Na/K-ATPase is the driving force for the reabsorption of Na^+^ and glucose as the primary ion transporter, also affects other cellular processes as a signal transducer. Compared with SGLT2 inhibitor's beneficial effects, the Na/K-ATPase and its signaling function exert some similar effects mainly through the kidney, such as natriuresis, blood pressure-lowering, and through the heart, such as reduction of cardiac hypertrophy and fibrosis. Even though the Na/K-ATPase signaling-mediated oxidant amplification loop was established in RPTs, the similarity of the Na/K-ATPase signaling cascades in both cardiac myocytes and renal proximal tubule cells suggests that this amplification loop might be shared in both cell types. For example, Ouabain-induced endocytosis of the Na/K-ATPase was first observed in HeLa cells, chick embryo heart cells, and Girardi heart cells (Aiton et al. 1981; Algharably et al. 1986; Cook 1982; Lamb and McCall 1971; Lamb and Ogden 1982). Since the signaling function of the Na/K-ATPase is redox-sensitive, a balanced ROS environment would be more beneficial. The possibility that the Na/K-ATPase and its signaling affect SGLT2 function, and vice versa, worth exploring.
